# CREB5 Promotes the Proliferation of Neural Stem/Progenitor Cells in the Rat Subventricular Zone via the Regulation of NFIX Expression

**DOI:** 10.3390/cells14161240

**Published:** 2025-08-12

**Authors:** Tao Yu, Hanyue Zhang, Chuang Zhang, Guorui Ma, Tu Shen, Yan Luan, Zhichao Zhang

**Affiliations:** Institute of Neurobiology, School of Basic Medical Sciences, Xian Jiaotong University Health Science Center, Xi’an 710061, China

**Keywords:** rat neural stem/progenitor cells, subventricular zone, CREB5, proliferation, NFIX

## Abstract

Neural stem/progenitor cells (NSPCs) in the subventricular zone (SVZ) of the central nervous system (CNS) are critical for tissue repair following injury or disease. These cells retain the capacity to proliferate, migrate, and differentiate into neurons, astrocytes, and oligodendrocytes, making them a promising therapeutic target for neurodegenerative disorders and traumatic injuries. However, the molecular mechanisms regulating their proliferation remain incompletely understood. This study investigates the role of cAMP responsive element-binding protein 5 (CREB5) in the proliferation of rat SVZ-derived NSPCs and elucidates its regulatory mechanism. Using RNA interference, we demonstrated that CREB5 knockdown significantly reduced cell viability, neurosphere formation capacity, and the number of proliferating cells (BrdU- and Ki-67-positive cells) both in vitro and in vivo. In contrast, CREB5 overexpression played opposing roles in cell proliferation. Additionally, alteration of CREB5 expression did not affect apoptosis, as assessed by TUNEL staining, indicating a specific role in proliferation rather than in cell death. Mechanistically, we identified Nuclear Factor One X (NFIX) as a transcriptional target of CREB5. CREB5 binds to the AP-1 site in the NFIX promoter, enhancing its expression. CREB5 knockdown inhibited NFIX expression, while CREB5 overexpression exerted the opposite function. ChIP and luciferase reporter assays further confirmed that CREB5 directly regulates NFIX promoter activity. More importantly, alteration of NFIX expression could reverse the effect of CREB5 on NSPC proliferation. These findings highlight CREB5 as a key regulator of NSPC proliferation through its interaction with NFIX, providing a potential therapeutic target for stem cell-based treatments of CNS disorders.

## 1. Introduction

Neural stem/progenitor cells (NSPCs) persist throughout life in mammals, where they differentiate into neurons, astrocytes, and oligodendrocytes within the central nervous system (CNS). These cells reside in two specialized regions: the subventricular zone (SVZ), which lines the lateral ventricle walls, and the subgranular zone (SGZ) in the hippocampal dentate gyrus [1]. The SVZ is the primary neurogenic region in the postnatal brain [2]. Previous studies demonstrated that NSPCs within the SVZ can be activated by various pathological stimuli, including stroke and traumatic injury, thereby contributing to CNS repair following damage [3]. Therefore, understanding the mechanisms that regulate NSPC proliferation is crucial for therapeutic strategies.

The gene encoding cAMP responsive element-binding protein 5 (CREB5), also referred to as CREB-BPA, is a member of the CREB protein family [4]. This protein interacts with cAMP response elements via its bZIP DNA-binding and zinc-finger domains, thereby acting as a CRE-dependent transcriptional activator [5]. CREB5 serves as a regulator in various biological processes, such as cartilage formation, embryonic development, and dopamine receptor-related neuroplasticity [6,7,8]. Notably, a growing number of studies have reported that CREB5 is strongly associated with the regulation of cell proliferation. Overexpression of CREB5 enhanced the invasiveness and metastatic potential of colorectal cancer cells, whereas silencing CREB5 decreased these capabilities [9]. In metastatic castration-resistant prostate cancers (mCRPCs), CREB5 can interact with FOXA1 and selectively enhance the interaction of AR with target genes critical for proliferation and survival [10]. Although the function of CREB5 in NSPCs remains largely unknown, previous research has shown that the phosphorylated form of CREB is enriched and specifically localized within the neurogenic zones of the adult mouse brain and that it plays a critical role in neuronal survival and synaptic plasticity [11,12,13,14]. Based on these findings, it is plausible to hypothesize that CREB5 plays a role in regulating the proliferation of NSPCs.

This study aimed to elucidate the role of CREB5 in NSPC proliferation and its regulatory mechanisms. First, NSPCs from the subventricular zone of postnatal day 7 (P7) rats were cultured, and CREB5 expression was evaluated in these cells. Cell proliferation was subsequently evaluated using BrdU and Ki-67 staining after modulating CREB5 expression in vivo and in vitro. We then analyzed NFIX expression and its interaction with CREB5 through mechanistic studies. Notably, altering the NFIX expression eliminated CREB5-mediated proliferative effects in rat NSPCs. These findings offer new insights into the role of CREB5 in NSPC proliferation and suggest a potential target for the development of stem cell-based therapies for central nervous system disorders.

## 2. Materials and Methods

### 2.1. Rats

The Sprague Dawley rats utilized in this study were procured from the Experimental Animal Center of Xi’an Jiaotong University. The Institutional Animal Care and Use Committee of Xi’an Jiaotong University granted approval for all the experimental protocols (Approval No. XJTUAE2023-445). The rats were housed under standard conditions, including a 12-h light/dark cycle, ad libitum access to food and water, and an ambient temperature maintained at 20–24 °C. Every effort was made to minimize animal suffering and to use the smallest possible number of animals in accordance with ethical guidelines.

### 2.2. Primary Culture of SVZ-Derived NSPCs

P7 rats were anesthetized with 5% isoflurane and euthanized using 65% CO_2_. SVZ tissues were then dissected and chopped into small pieces in ice-cold DMEM/F12 medium (Thermo Fisher Scientific, Waltham, MA, USA, 11320033) and subsequently incubated with ACCUTASE™ (STEMCELL Technologies, Vancouver, BC, Canada, 07920) at 37 °C for 5 min. The tissues were then dissociated into a single cell, followed by filtration through a 40 μm cell strainer (Corning Falcon, New York, NY, USA, Cat. No. 352340). The filtered suspension was centrifuged at 1000× *g* for 3 min at 4 °C and seeded at a density of 20,000 cells/mL in non-adhesive T75 flasks containing complete medium (serum-free DMEM/F12 supplemented with 1% N-2, 2% B-27™, 20 ng/mL EGF, and 10 ng/mL bFGF). All reagents were sourced from Thermo Fisher Scientific. The cells were incubated at 37 °C in a CO_2_ incubator (Memmert, Schwabach, Germany, ICO150), with EGF and bFGF (10 ng/mL) replenished every other day. Neurospheres were passaged twice to remove debris. Neurospheres and single NSPCs were plated onto poly-D-lysine (PDL)-coated culture plates or coverslips. To assess the differentiation potential of NSPCs, cells were cultured on PDL-coated coverslips in a medium devoid of growth factors and supplemented with 1% fetal bovine serum (Thermo Fisher Scientific).

### 2.3. siRNA-Mediated CREB5 Knockdown

The siRNAs (si-CREB5-1, siRNA ID: s178974; si-CREB5-2, siRNA ID: RSS365325; si-CREB5-3, siRNA ID: RSS365327; si-NFIX-1, siRNA ID: s135365; si-NFIX-2, siRNA ID: RSS340203; si-NFIX-1, siRNA ID: RSS372785) were purchased from Thermo Fisher, while the scrambled siRNA negative control was procured from GenePharma (Shanghai, China). Single neural stem/progenitor cells (NSPCs) were cultured on poly-D-lysine (PDL)-coated coverslips and transfected with siRNA using LipofectamineTM 3000 (L3000001, Thermo Fisher). Six hours post-transfection, the transfection efficiency was evaluated using an inverted fluorescence microscope (DMI3000B, Leica, Wetzlar, Germany). The following day, CREB5 expression levels were assessed via Western blot analysis. Cells were subsequently used for further experiments 24 h after siRNA transfection. All experiments were conducted in triplicate and independently repeated at least three times.

### 2.4. Cell Viability Assay

Cell viability was assessed using the Cell Counting Kit-8 (CCK-8; C008, 7Sea, Shanghai, China). Following siRNA transfection, NSPCs were plated at a density of 5000 cells per well in PDL-coated 96-well plates and incubated for 24 h prior to experimentation. At the end of each treatment, 20 µL of CCK-8 solution was added to each well, and the plates were incubated for an additional 2 h. Absorbance was measured at 450 nm using a multimode microplate spectrophotometer (Epoch; BioTek, Winooski, VT, USA). Three replicates were performed for each treatment, and data were expressed as the average of at least three independent experiments.

### 2.5. Sphere Formation Assay

Following siRNA transfection, 8000 cells per well were seeded in a 24-well plate. The number and diameter of neurospheres were evaluated on days 1, 2, and 3 using an inverted microscope (Olympus, Tokyo, Japan, CKX41). The day after transfection was designated as day 0. Images were analyzed with ImageJ 3.5 software to quantify neurospheres with a diameter of at least 30 μm per well. All experiments were replicated three times.

### 2.6. Lentiviral Transfection

Lentiviral vectors for CREB5 inhibition (KD-CREB5, 7 × 10^7^ TU/mL), NFIX overexpression (Ov-NFIX, 1 × 10^8^ TU/mL), and a control (mock, 2 × 10^8^ TU/mL) were purchased from GenePharma (Shanghai, China). Ten-week-old rats were anesthetized with 5% isoflurane and maintained under 2.5% isoflurane. The rats were placed in a stereotactic frame (RWD Life Science, Johannesburg, South Africa, 69100), and a longitudinal incision was made along the skull midline to expose the bregma. A small hole was drilled, and the lentivirus was stereotaxically injected into the right dentate gyrus (DG) using the following coordinates: anterior–posterior, 1.08 mm anterior to the bregma; medial–lateral, 1.12 mm; and dorsal–ventral, 4.40 mm below the skull surface. A total of 2 μL of the lentiviral solution was injected into the lateral ventricle at a flow rate of 200 nL/min using a microsyringe pump (KD Scientific Legato™ 130, Holliston, MA, USA). The needle was left in place for 5 min to ensure complete delivery before being slowly withdrawn. For in vitro experiments, rat SVZ-derived NSPCs were transfected with si-CREB5-1 and subsequently infected with either Ov-NFIX or mock viruses the following day. Cell proliferation was assessed by immunostaining after 5 days.

### 2.7. Immunostaining

Rats were anesthetized with Xylazine (0.12 mL/100 g) and then perfused transcardially with 250 mL of normal saline, followed by 400 mL of 4% paraformaldehyde (PFA). The brains were excised, post-fixed overnight in 4% PFA at 4 °C, and cryoprotected in 30% sucrose for at least 3 days at 4 °C. Coronal sections (16 μm thick) were cut using a freezing microtome (RWD), with one section out of every three mounted on adhesive slides and air-dried. For in vitro experiments, cells were fixed with 4% PFA for 30 min at room temperature (RT) at the end of treatment. After three washes with 0.01 M PBS, slides and coverslips were permeabilized with 0.1% Triton X-100 for 15 min, followed by blocking with a buffer containing 5% bovine serum albumin and 5% horse serum for 2 h at RT. For BrdU staining, samples were pretreated with 2N HCl at 37 °C for 40 min and neutralized in 0.1 M borate buffer (pH 8.5) for 15 min. Primary antibodies (Table S1) were applied, and samples were incubated overnight at 4 °C. For immunohistochemical staining, biotinylated secondary antibodies (Thermo Fisher) were incubated at RT and detected using an avidin–biotin complex peroxidase kit (Vector Laboratories, Newark, CA, USA), followed by development with diaminobenzidine (DAB, Vector Laboratories). For immunofluorescence staining, primary antibodies were visualized using appropriate secondary antibodies. Nuclei were stained with DAPI or PI, and images were captured using a fluorescence microscope (Olympus BX51 + DP71). Image analysis was performed using Image-Pro Plus 6.0 to automatically count positively stained nuclei. All experimental procedures were conducted in triplicate.

### 2.8. Western Blot

Brain tissues and cells were harvested following treatments and lysed in RIPA buffer (Abcam, ab156034) supplemented with Protease Inhibitor Cocktail (Roche, Basel, Switzerland, 11697498001) for 15 min on ice. The lysates were then sonicated using a Sonics device (Newtown, CT, USA). The lysates were then centrifuged at 12,000× *g* for 10 min at 4 °C to separate the supernatant, which was assayed for protein concentration using a BCA kit (Thermo Fisher Scientific, 23227). Proteins were denatured by boiling with loading buffer and loaded onto 12% SDS-PAGE gels (20–40 μg per lane). Following electrophoresis, proteins were transferred to PVDF membranes (Bio-Rad, Hercules, CA, USA, 1620177). The membranes were blocked with 5% non-fat milk in TBST and incubated overnight with primary antibodies (Table S1) at 4 °C. After washing with TBST, the membranes were incubated with horseradish peroxidase-conjugated secondary antibodies for 2 h at room temperature. After thorough rinsing, immunoreactive bands were detected using SuperSignal™ West Pico PLUS Chemiluminescent Substrate (Thermo Fisher Scientific, 34580) and Fuji X-ray film (Fujifilm Corporation, Tokyo, Japan). Band images were captured with a gel imaging system (G:Box, Syngene, UK) and analyzed using ImageJ 3.5 software. β-Actin was used as the internal control to normalize target protein levels. All experiments were performed in triplicate.

### 2.9. qRT-PCR

Total RNA was extracted from cultured NSPCs using the Trizol reagent (Invitrogen, Carlsbad, CA, USA) and reverse-transcribed into cDNA with the RevertAid First Strand cDNA Synthesis Kit (Thermo Fisher Scientific). The cDNA synthesis utilized a combination of oligo(dT) and random primers. qRT-PCR was conducted using GoTaq qPCR Master Mix (Roche, Germany) on an iQ5 Real-Time PCR Detection System (Bio-Rad, USA). The primer sequences were synthesized by TaKaRa (Dalian, China)and are listed below. CREB5: Forward: GCAGCAAGTCATCCAGCATA; Reverse: TGTACGGCATGCTACAGCTC. GAPDH: Forward: GACAACTTTGGCATCGTGGA; Reverse: ATGCAGGGATGATGTTCTGG. Relative fold changes in target gene expression were calculated using the 2^−ΔΔCt^ method, with GAPDH serving as the housekeeping gene for normalization.

### 2.10. Chromatin Immunoprecipitation (ChIP)-qPCR

Briefly, 200 μL of single-cell suspensions (1 × 10^7^ cells/mL) were crosslinked with 1% formaldehyde for 10 min and then quenched with glycine (final concentration 125 mM) for 5 min. After two washes with ice-cold PBS, cells were lysed in 300 μL of lysis buffer and sonicated for 3 cycles of 30 s each using a VCX 500 (SONICS, Newtown, CT, USA). The lysate was diluted and incubated with Dynabeads™ Protein A beads (Invitrogen), which were pre-bound with 2 μg of antibody against CREB5 or IgG (Abcam, Cambridge, UK). The immune complexes were incubated at 4 °C, rotating at 20 rpm on a Tube Revolver Rotator for 12 h. The chromatin–antibody complexes were washed four times with ice-cold RIPA buffer, rinsed with ChIP elution buffer (containing 50 μg RNase A), and heated at 37 °C, 1200 rpm on a Thermomixer (Eppendorf, Hamburg, Germany) for 1 h. Subsequently, the samples were incubated with 2 μL of Proteinase K (New England Biolabs, Ipswich, MA, USA) at 65 °C for 4 h. An input control was processed concurrently. ChIP DNA was purified using phenol–chloroform extraction, precipitated with ethanol, and resuspended in 20 μL of EB buffer (Qiagen, Singapore). DNA analysis was conducted via qRT-PCR with AP-1 binding site primers. Forward: GGCTCTTGGCTGAGATTCAC; Reverse: GCCCCTCTCCTCATCACTCT. The percentage of input DNA was calculated by comparing the immunoprecipitated DNA with the input DNA.

### 2.11. Luciferase Reporter Assay

To amplify the NFIX promoter fragment, rat genomic DNA (Merck Millipore, Burlington, MA, USA, 69238) was used as the template. The NFIX promoter region was PCR-amplified and subsequently ligated into the pGL3-Basic vector (Promega, WI, USA) according to the manufacturer’s instructions. HEK293T cells were transfected in 24-well plates at 70% confluence using Lipofectamine 2000 (Thermo Scientific). After 48 h, luciferase activity was measured using the Dual-Luciferase Reporter Assay System (Promega, WI, USA) and normalized to Renilla luciferase expression. All experiments were performed in triplicate.

### 2.12. Statistical Analysis

Statistical analyses were performed using GraphPad Prism 5.0 (San Diego, CA, USA). Prior to comparative analyses, data were evaluated for normality and homogeneity of variances. One-way ANOVA was used to determine statistical significance, followed by Fisher’s PLSD test for pairwise comparisons. The Kolmogorov–Smirnov test was applied to assess normality and variance equality. Results are expressed as mean ± standard deviation, with *p* < 0.05 denoting statistical significance.

## 3. Results

### 3.1. CREB5 Suppression Reduces Cell Viability and Sphere Formation Ability in Cultured Rat SVZ NSPCs

**Figure 1:**
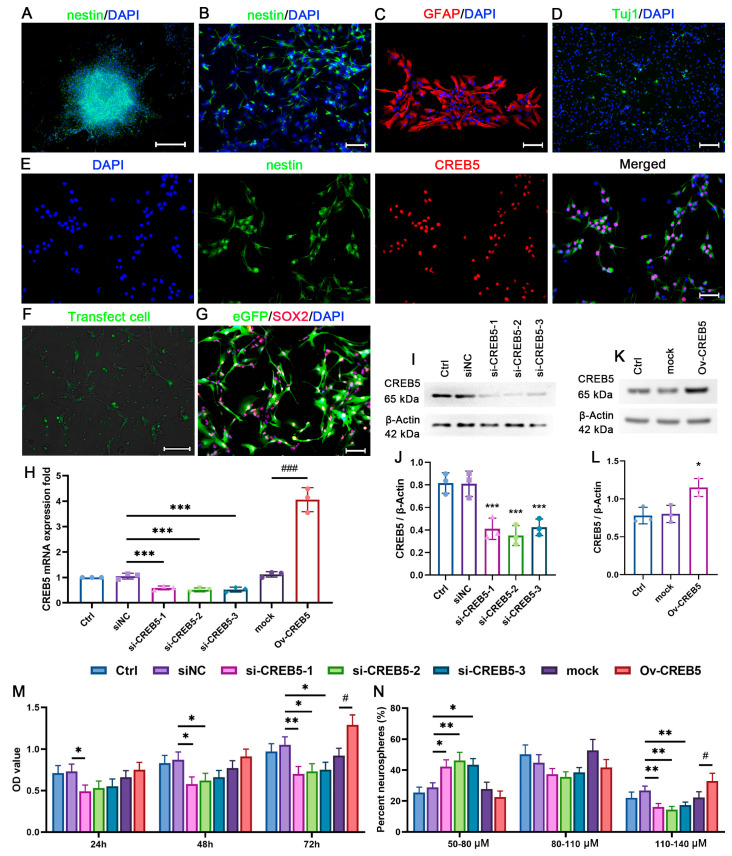
CREB5 is involved in the regulation of cell viability and neurosphere formation capacity in cultured rat SVZ-derived NSPCs. Neurospheres (**A**) and individual NSPCs (**B**) were plated onto PDL-coated coverslips in complete medium. Immunostaining revealed that the majority of these cells expressed nestin. NSPCs were incubated in a natural differentiation medium for three days. The differentiation capacity of NSPCs was assessed by immunostaining for GFAP (**C**) and Tuj1 (**D**). (**E**) CREB5 expression was co-localized with nestin, as observed through double immunofluorescence staining. NSPCs were transfected with either siRNA (**F**) or lentivirus (**G**). Subsequently, CREB5 expression was assessed by qRT-PCR (**H**) and Western blot (**I**,**K**), respectively. (**J**,**L**) Data are presented as the mean ± standard deviation from three independent experiments (n = 3). *** *p* < 0.001 versus the siNC group; ^###^ *p* < 0.001 versus the mock group. (**J**). * *p* < 0.05 versus the mock group (**L**). Cell viability (**M**) and neurosphere diameter (**N**) were measured at different time points post-transfection. Data are presented as the mean ± standard deviation of three independent experiments (n = 3). * *p* < 0.05; ** *p* < 0.01 versus the siNC group; ^#^ *p* < 0.05 versus the mock group. Scale bars: A = 200 μm; B, C, E, and F = 50 μm; D = 100 μm. siNC, negative control siRNA; mock, empty lentivirus.

Primary NSPCs were isolated from the SVZ of P7 rat, and lots of neurospheres were observed in the culture medium after culturing for 3 days. Both neurospheres and individual NSPCs grew as adherent cultures. Immunofluorescence staining for nestin showed that the majority of these cells exhibited nestin positivity (Figure 1A,B). Following a 3-day culture in the natural differentiation medium, both Tuj1-positive (Figure 1C) and GFAP-positive (Figure 1D) cells were detected. These results collectively confirm that the cultured cells are primary SVZ-derived NSPCs. Moreover, double immunostaining showed that CREB5 was co-localized with the NSPC marker nestin (Figure 1E). To investigate the role of CREB5 in NSPCs, we modulated its expression using RNA interference (RNAi) and CREB5-overexpressing lentiviral vectors. Following transfection, both CREB5-specific siRNA (Figure 1F) and CREB5-overexpressing lentiviral vectors (Figure 1G) were efficiently delivered into cells, as confirmed by fluorescence microscopy. qRT-PCR and Western blot analyses demonstrated that the three siRNAs effectively suppressed CREB5 expression at the mRNA and protein levels, respectively (Figure 1H–J), whereas lentiviral-mediated overexpression elevated CREB5 expression (Figure 1K,L). Then, a preliminary evaluation of CREB5’s effect on NSPC proliferation was conducted using the CCK-8 and sphere formation assays. CREB5 knockdown significantly diminished cell viability and reduced neurosphere diameter compared with those in the siNC control group. Conversely, CREB5 overexpression enhanced both proliferative capacity and neurosphere size (Figure 1M,N). These findings suggest a possible association between CREB5 expression and the regulation of proliferation in NSPCs derived from rat SVCs.

### 3.2. Modulation of CREB5 Expression Regulates NSPC Proliferation in the Rat SVZ

**Figure 2:**
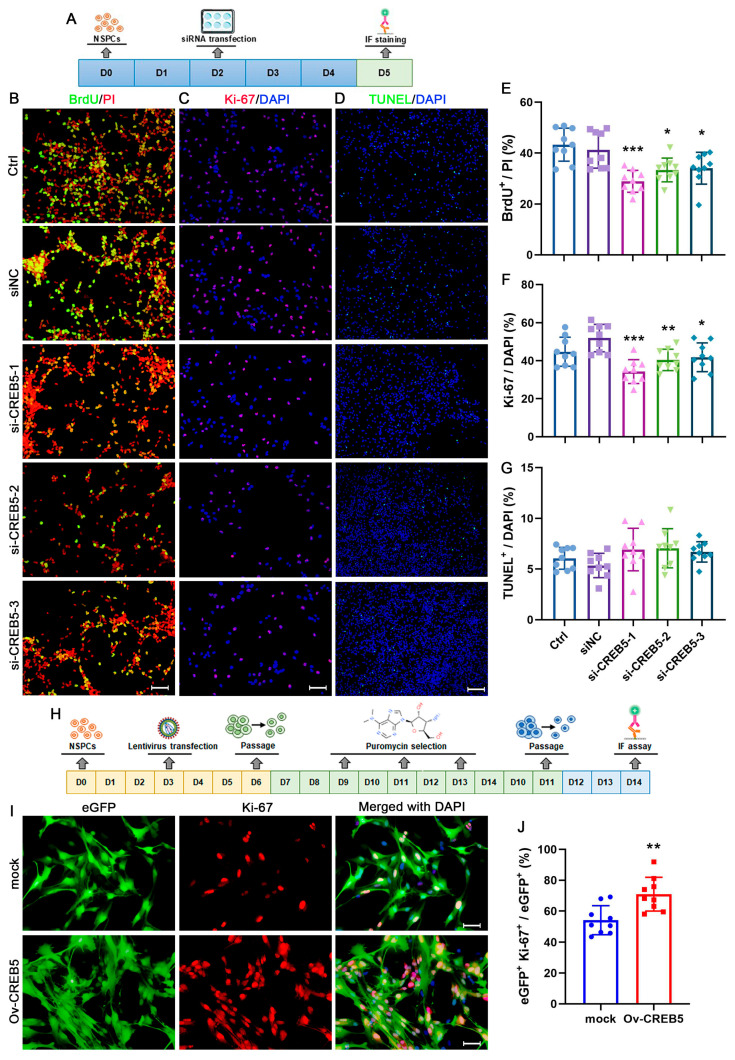
CREB5 overexpression promotes the proliferation of cultured rat SVZ-derived NSPCs. (**A**) Flowchart of siRNA transfection experiments. Following a 3-day culture period, the presence of BrdU-positive (**B**) and Ki-67-positive (**C**) cells was determined using immunostaining. The results are reported as percentages of either PI-stained (**E**) or DAPI-stained (**F**) cells. Data are presented as the mean ± standard deviation of nine independent experiments (n = 9). * *p* < 0.05, ** *p* < 0.01, *** *p* < 0.001 versus the siNC group. (**D**,**G**) Apoptotic NSPCs were identified using TUNEL staining. Data are presented as mean ± standard deviation from nine independent experiments (n = 9). (**H**) Cells were infected with lentivirus and subsequently selected using puromycin. (**I**) Cell proliferation was assessed via Ki-67 staining. (**J**) Results are expressed as percentages of DAPI-stained cells. Data represent the mean ± standard deviation from nine independent experiments (n = 9). ** *p* < 0.01 versus the mock group. Scale bars: B, C, and I = 50 μm; D = 100 μm.

To clarify the impact of CREB5 on the proliferation of rat SVZ NSPCs, a BrdU incorporation assay was performed to assess cell proliferation (Figure 2A). The results showed that transfection with siNC had minimal impact on BrdU staining outcomes. In contrast, treatment with si-CREB5-1 led to a significant reduction in the proportion of BrdU-positive cells, decreasing from 41.3% ± 6.5 to 28.9% ± 4.3 (*p* = 0.0006). Similarly, both si-CREB5-2 and si-CREB5-3 treatments exhibited comparable effects on NSPC proliferation (Figure 2B,E). Subsequently, Ki-67 immunostaining was used to further assess cell proliferation. As anticipated, the proportion of Ki-67-positive cells significantly decreased following si-CREB5-1 treatment (from 51.9% ± 7.2 to 34.3% ± 6.3, *p* < 0.0001). This reduction was also evident in the si-CREB5-2/3 treatment groups (Figure 2C,F). To exclude the possibility that CREB5 affects NSPC proliferation through apoptosis, TUNEL staining was performed to identify apoptotic cells. No significant differences were observed among the treatment groups, indicating that the effects on proliferation were not mediated by apoptosis (Figure 2D,G). Then, cells were stably infected with either an empty lentiviral vector (mock) or a CREB5-overexpressing lentiviral vector (Ov-CREB5; Figure 2H). In the mock group, the proportion of eGFP+Ki67+ cells was 54.2% ± 9.3%. CREB5 overexpression significantly elevated this proportion compared with that in the mock group (*p* = 0.0029, Figure 2I,J).

**Figure 3:**
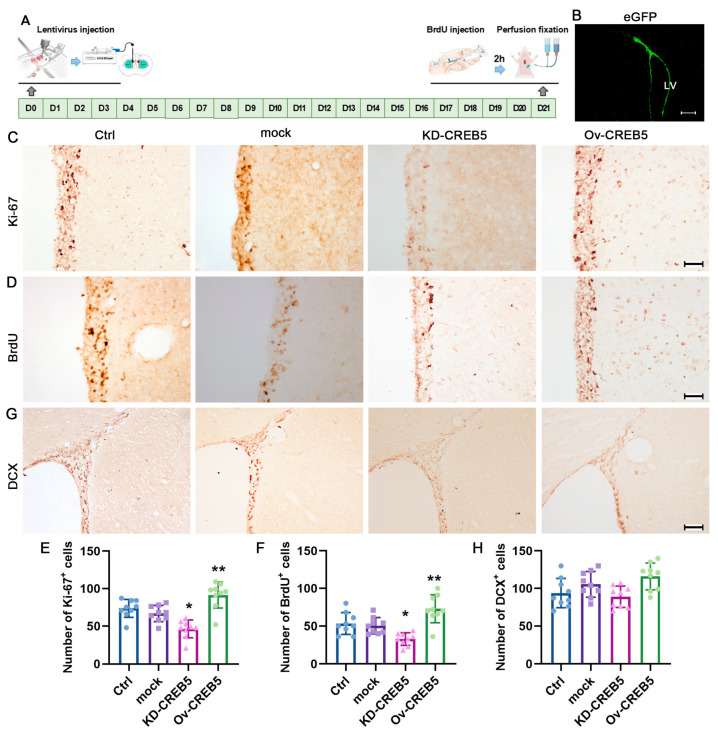
CREB5 overexpression promotes NSPC proliferation in the rat SVZ. (**A**) Flowchart of the in vivo experiments. (**B**) Lentiviruses targeting CREB5 knockdown (KD-CREB5), CREB5 overexpression (Ov-CREB5), or empty lentivirus (mock) were injected into the rat lateral ventricle, and eGFP-positive cells were visualized three weeks post-injection. NSPC proliferation was evaluated using Ki-67 (**C**) and BrdU (**D**) immunostaining, while NSPC differentiation within the SVZ was assessed via DCX immunostaining (**G**). (**E**,**F**,**H**) Data are presented as the mean ± standard deviation of nine independent experiments (n = 9). * *p* < 0.05, ** *p* < 0.01 versus the mock group. Scale bars: B = 200 μm; C, E, and G = 50 μm. mock, empty lentivirus.

To further investigate the role of CREB5 in NSPC proliferation, we conducted an in vivo study using rats. Among the siRNA constructs tested, si-CREB5-1 exhibited the most potent inhibitory effect and was consequently selected for lentiviral packaging driven by the nestin promoter. Three weeks after virus injection, eGFP-positive cells were predominantly localized in the SVZ area (Figure 3A,B). Proliferating cells were then assessed by Ki-67 and BrdU staining. Compared with the empty lentivirus (mock), CREB5 knockdown (KD-CREB5) significantly reduced the number of Ki-67- (from 66.8% ± 10.8 to 46.3% ± 11.8, *p* = 0.0126) and BrdU- (from 50.4% ± 10.8 to 32.8% ± 8.3, *p* = 0.0448) positive cells. In contrast, CREB5 overexpression (Ov-CREB5) had the opposite effects (Figure 3C–F). To rule out the potential effect of CREB5 on NSPC differentiation, we performed DCX immunostaining. The results showed that alteration of CREB5 expression had a minimal effect on the number of DCX-positive cells (Figure 3G,H). These results strongly suggest that CREB5 plays a critical role in regulating the proliferation of NSPCs in the rat SVZ.

### 3.3. CREB5 Regulates NFIX Expression in Rat SVZ-Derived NSPCs

**Figure 4:**
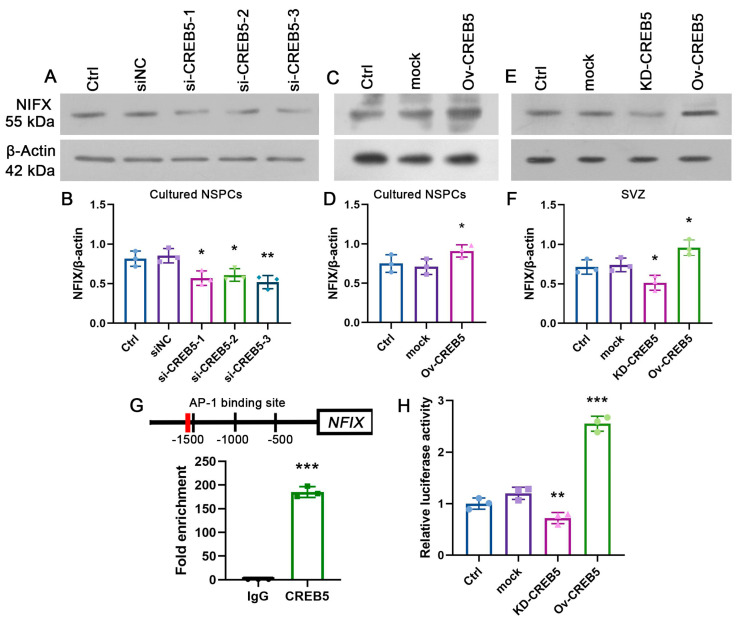
CREB5 is involved in the regulation of NFIX expression. (**A**,**B**) Neural stem/progenitor cells (NSPCs) were seeded on poly-D-lysine (PDL)-coated coverslips and transfected with siRNAs. NFIX expression was subsequently evaluated by Western blot analysis. The ratio of CREB5 to β-actin was employed to plot the band intensity. Data are presented as the mean ± standard deviation of three independent experiments (n = 3). * *p* < 0.05, ** *p* < 0.01 versus the siNC. (**C**,**E**) NSPCs were transduced with lentivirus in cultured NSPCs (**C**) and SVZ region (**E**), followed by Western blot analysis of NFIX expression. (**D**,**F**) The ratio of CREB5 to β-actin was employed to plot the band intensity. Data are presented as the mean ± standard deviation of three independent experiments (n = 3). * *p* < 0.05 versus the mock group. (**G**) ChIP-qPCR was performed to assess CREB5 enrichment at the AP-1 binding site within the NFIX promoter. The values are presented as the mean ± standard deviation of three independent experiments (n = 3). *** *p* < 0.001 versus the IgG group. (**H**) Cultured NSPCs were transduced with lentivirus encoding CREB5 knockdown (KD-CREB5), CREB5 overexpression (Ov-CREB5), or empty vector (mock). Dual-luciferase reporter assays validated the interrelationship between NFIX and CREB5. The values are presented as the mean ± standard deviation of three independent experiments (n = 3). ** *p* < 0.01, *** *p* < 0.001 versus the mock group.

NFIX, a member of the Nuclear Factor I (NFI) transcription factor family, plays a pivotal role in CNS development [15]. The specific knockout of NFIX in NSPCs significantly impairs cell proliferation [16]. A previous study revealed that CREB5 binds to the NFIX promoter, thereby enhancing its expression [17]. Consequently, it is reasonable to speculate that CREB5 regulates NSPC proliferation through binding to the NFIX promoter and through subsequent effects on its expression. To confirm this hypothesis, we collected cultured NSPCs and subventricular zone (SVZ) tissues and measured NFIX expression using Western blot analysis. The results revealed that CREB5 knockdown markedly reduced NFIX expression both in vitro and in vivo (Figure 4A,B,E,F). In contrast, CREB5 overexpression significantly improved the level of NFIX (Figure 4C,D). Since CREB5 regulates gene transcription by binding to AP-1 sites [18], we performed chromatin immunoprecipitation (ChIP) assays to investigate its interaction with the NFIX promoter. These assays demonstrated that CREB5 directly interacts with the AP-1 motif within the NFIX promoter region (Figure 4G). Furthermore, luciferase reporter assays revealed a significant reduction in NFIX promoter activity following CREB5 knockdown in SVZ-derived NSPCs (0.6-fold, *p* = 0.0057). In contrast, CREB5 overexpression increased luciferase activity 2.13-fold (*p* < 0.0001; Figure 4H). Collectively, these findings indicate that CREB5 transcriptionally regulates NFIX expression via direct interaction with the AP-1 site in its promoter.

### 3.4. CREB5 Influences Proliferation of Rat SVZ-Derived NSPCs by Regulating NFIX Expression

**Figure 5:**
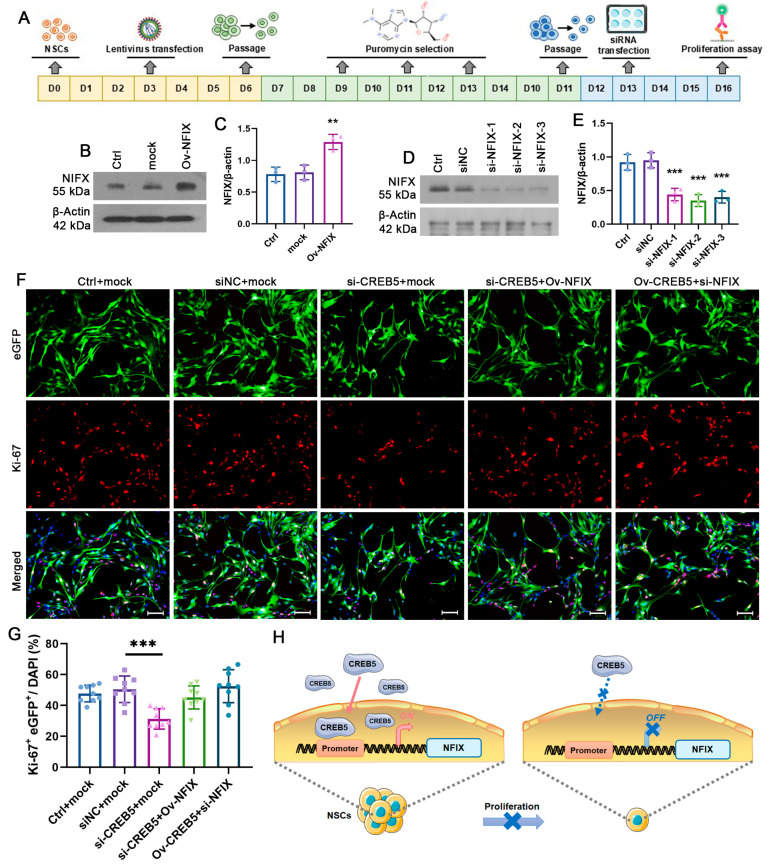
Alteration of NFIX expression attenuates the proliferative effect of CREB5 in NSPCs. (**A**) Flowchart of NSPC transfection experiments. The cultured NSPCs were infected with lentiviruses encoding NFIX (Ov-NFIX) or empty vectors (mock), respectively. Following puromycin selection, the NFIX expression was detected by Western blot. (**B**,**C**) The intensity of bands from representative blots was quantified and expressed as the NFIX to β-actin ratio. Data are presented as the mean ± standard deviation of three independent experiments (n = 3). ** *p* < 0.01 versus the mock group. (**D**) NSPCs were transfected with NFIX-targeting siRNAs or siNC for 3 days. NFIX protein levels were then quantified by Western blotting. (**E**) Data are presented as the mean ± standard deviation from three independent experiments (n = 3). *** *p* < 0.001 versus the siNC group. (**F**,**G**) After infection with Ov-NFIX or mock, cells were transfected with non-targeting siRNA (siNC) or three CREB5-specific siRNAs (si-CREB5) for 72 h. Proliferating cells were quantified via Ki-67 staining. Data are presented as the mean ± standard deviation of nine independent experiments (n = 9). *** *p* < 0.001 versus the siNC +mock group; ** *p* < 0.01 versus the si-CREB5 +mock group. Scale bars = 100 μm. (**H**) The illustration demonstrates the mechanism by which CREB5 inhibition modulates the proliferation of NSPCs derived from the rat SVZ. CREB5 facilitates the expression of NFIX by binding to the AP-1 site within its promoter region, thereby promoting NSPC proliferation (**left** panel). Conversely, CREB5 knockdown leads to a reduction in NFIX expression, which in turn impairs cell proliferation (**right** panel).

To assess whether CREB5 influences NSPC proliferation by modulating NFIX expression, we overexpressed NFIX using a lentiviral vector (Ov-NFIX) or silenced it via RNA interference (Figure 5A). Western blot analysis demonstrated a marked increase in NFIX protein levels following transfection and selection (Figure 5B,C). Additionally, all three NFIX-targeting siRNAs effectively suppressed NFIX expression (Figure 5D,E). Ki-67 immunostaining was then employed to assess cellular proliferation. No significant difference in proliferative cells was detected between the Ctrl+mock (47.7% ± 5.4) and siNC+mock (50.5% ± 8.6) groups. However, the si-CREB5+mock group exhibited a significant reduction in Ki-67-positive cells compared with the siNC+mock group (from 50.5% ± 8.6 to 31.3% ± 6.6, *p* < 0.0001). Notably, NFIX overexpression rescued this proliferative deficit, restoring cell proliferation to levels comparable with those in siNC+mock groups. Conversely, NFIX inhibition abolished the proliferative effect of CREB5 overexpression in cultured NSPCs (Figure 5F,G). These findings demonstrate that CREB5 is a critical regulator of rat NSPC proliferation, acting through modulation of NFIX expression.

## 4. Discussion

NSPCs have emerged as a promising avenue for repairing CNS damage due to their ability to differentiate into various cell types, including neurons, astrocytes, and oligodendrocytes. This multipotency allows NSPCs to potentially replace lost or damaged cells, thereby contributing to the restoration of CNS function [19,20]. The therapeutic potential of NSPCs is particularly significant in the context of neurodegenerative diseases and traumatic injuries, where the regeneration of neural tissue is crucial for recovery. Endogenous NSPCs, located in neurogenic niches such as the subventricular zone, can be activated following injury to proliferate, migrate to the site of damage, and differentiate into the necessary cell types [21]. However, the innate response of these cells is often insufficient for complete functional recovery, necessitating interventions to enhance their activation and efficacy. Understanding the molecular mechanisms that regulate NSPC behavior is crucial for developing strategies to harness their full regenerative potential [22]. In this study, we demonstrated that CREB5 is expressed in rat SVZ NSPCs. Suppression of CREB5 expression significantly reduced the capacity for neurosphere formation and inhibited NSPC proliferation without affecting differentiation or apoptosis. In contrast, CREB5 overexpression enhanced NSPC proliferation. Furthermore, CREB5 was found to bind to NFIX, a key regulator of NSPC proliferation, and CREB5 inhibition markedly decreased NFIX expression. Vice versa, CREB5 overexpression promoted NFIX expression. Alteration of NFIX expression eliminated the suppression effect on NSPC proliferation following CREB5 inhibition. These findings strongly suggest that CREB5 promotes NSPC proliferation by binding to the NFIX promoter and enhancing its expression (Figure 5H). However, it needs to be noted that CREB5 is highly or abnormally expressed in numerous tumors [23,24,25]. Abnormal NSPC proliferation, characterized by the excessive and uncontrolled division of NSPCs, may contribute to the formation of glioblastoma stem cells (GSCs) [26,27]. Further studies are essential to comprehensively evaluate the biosafety implications of CREB5. Moreover, a limitation of this study is the exclusive use of rat NSPCs, which may restrict the clinical relevance of our findings. Future work should therefore validate CREB5 function in human NSPCs.

CREB, or cAMP response element-binding protein, is a key player in how cells respond to external signals. It acts as a transcription factor, meaning it helps turn on or off specific genes inside the nucleus of cells. When activated, it can influence various cell behaviors, such as how neurons form memories or survive under stress [28]. CREB is activated primarily through phosphorylation, a process in which a phosphate group is added to the protein, specifically at the Ser133 site. This is often achieved by an enzyme called protein kinase A (PKA), which is activated by cAMP. Other kinases, like calcium/calmodulin-dependent protein kinases, can also play a role, depending on the cell type and signal [29]. Once phosphorylated, CREB binds to DNA at specific sites called cAMP response elements (CREs), with the sequence TGACGTCA, to start or stop gene transcription [30]. CREB regulates a wide range of cell processes, especially in neurons. It is crucial for long-term memory formation, neuronal plasticity (how neurons adapt and change), and survival. It controls genes like BDNF, which supports neuron growth, and c-fos, involved in cell response to stimuli [31]. Beyond the brain, CREB influences circadian rhythms, helping cells keep time with day–night cycles, and has roles in conditions like cancer and depression [32]. These findings suggest that, compared with other tissues and organs, the CREB family may play a more critical role in the CNS. In this study, the results demonstrated that CREB5 regulates the proliferation of rat SVZ NSPCs. Suppression of CREB5 significantly inhibited NSPC proliferation by reducing NFIX promoter binding and inhibiting its expression, and vice versa. Previous studies have shown that CREB phosphorylation and CRE-mediated gene expression are closely linked to neuroprotection, neurite outgrowth, memory formation, and neurodegenerative diseases [33,34]. Impaired CREB-mediated transcription results in reduced energy metabolism, which subsequently leads to neuronal death and affects life expectancy [35]. Decreased CREB phosphorylation diminishes transcriptional activity, impacting synaptic plasticity and ultimately leading to synapse loss [36]. Additionally, CREB-knockout mice exhibit a disconnection between locomotor activity and anxiety-like behaviors [37]. Therefore, it is plausible to hypothesize that beyond its role in regulating NSPC proliferation, CREB5 may also influence dendritic and axonal development, as well as neuronal survival. Further studies, particularly in vivo investigations, are essential to comprehensively elucidate the functions of CREB5 in NSPC proliferation and differentiation. While our findings demonstrate the promoting effects of CREB5 on neural stem cell proliferation, it is important to acknowledge that excessive stem cell proliferation may pose potential tumorigenic risks, as dysregulated stem cell self-renewal has been implicated in oncogenesis. Future therapeutic applications of CREB5 overexpression will require comprehensive long-term safety assessments, including dose–response evaluations and extended monitoring protocols to mitigate any potential oncogenic transformation.

NFIX, a pivotal member of the Nuclear Factor I (NFI) family of transcription factors, plays a multifaceted and critical role in orchestrating the delicate equilibrium between stem cell self-renewal and differentiation [38]. Its function extends beyond simple binary control; NFIX acts as a sophisticated rheostat, finely tuning the expression of a vast network of genes that govern stem cell fate [39]. Studies have demonstrated that NFIX directly regulates the transcription of genes crucial for maintaining pluripotency, such as those encoding key components of the Wnt, Notch, and Hedgehog signaling pathways [40]. For instance, NFIX has been shown to bind to the promoter regions of genes like *Nanog* and *Oct4*, essential for maintaining the pluripotent state of embryonic stem cells. Conversely, NFIX also actively participates in the differentiation process, ensuring a timely and orderly transition from pluripotency to lineage-committed cell types [16]. This seemingly contradictory function is explained by NFIX’s ability to both activate and repress gene expression, depending on the cellular context and the specific target gene. It achieves this through its interaction with a diverse array of co-factors and epigenetic modifiers [41]. In some instances, NFIX may recruit histone deacetylases (HDACs) to target gene promoters, leading to chromatin condensation and transcriptional repression, thus promoting differentiation [42]. In other cases, NFIX can interact with histone acetyltransferases (HATs), resulting in chromatin de-condensation and transcriptional activation of genes associated with specific lineages [43]. Our present study indicates that CREB5 suppression leads to a decrease in NFIX expression and NFIX overexpression, while overexpression of NFIX abolishes the inhibitory effect of CREB5 on NSPC proliferation. These findings indicate that CREB5 primarily influences NSPC proliferation through the regulation of NFIX expression. However, it should be noted that the impact of CREB5 on NSPCs is not solely mediated by its regulation of NFIX. As a transcriptional activator, CREB5 can interact with multiple regulators of stem cell maintenance and differentiation, including Fos [44], CTBP1 [45], and MLLT6 [44]. These genes are strongly implicated in NSPC proliferation, differentiation, and apoptosis [46,47,48]. Further research is necessary to clarify the regulatory mechanisms of CREB5 by integrating ChIP-seq and RNA-seq data, thereby offering a comprehensive understanding of the genome-wide regulatory network.

## 5. Conclusions

Our study showed that CREB5 is essential for regulating proliferation of rat SVZ-derived NSPCs. Its knockdown decreases cell viability, neurosphere formation, and BrdU/Ki-67 labeling, while overexpression produces the opposite effects. Mechanistically, CREB5 directly binds an AP-1 site in the NFIX promoter to enhance NFIX transcription. Modulating NFIX levels rescues or abolishes the proliferative changes induced by CREB5 alteration. This finding offers a potential target for stem cell-based therapies in central nervous system disorders.

## Data Availability

The data used and/or analyzed during the current study are available from the corresponding author on reasonable request.

## Supplementary Materials

The following supporting information can be downloaded at https://www.mdpi.com/article/10.3390/cells14161240/s1. Table S1: Antibodies used during the study.

## Abbreviations

NSPCs	neural stem/progenitor cells
SVZ	subventricular zone
CNS	central nervous system
CREB5	cAMP responsive element-binding protein 5
NFIX	nuclear factor one X
SGZ	subgranular zone
mCRPCs	metastatic castration-resistant prostate cancers
ChIP	chromatin immunoprecipitation
PKA	protein kinase A
HDACs	histone deacetylases
HATs	histone acetyltransferases
PFA	paraformaldehyde
