# Supplementary material for: CREB5 Promotes the Proliferation of Neural Stem/Progenitor Cells in the Rat Subventricular Zone via the Regulation of NFIX Expression
Source: Cells. 2025 Aug 12;14(16):1240. doi: 10.3390/cells14161240 (PMC12384836; doi:10.3390/cells14161240)
Supplement: Supplementary file 1 [file cells-14-01240-s001.zip › cells-3787055-supplementary materials.pdf]

Supplement Table 1 Antibodies used during the study

Antigen	Source and host species	Concentration	Catalog No.
anti-nestin	Invitrogen, rabbit monoclonal antibody	1:200	PA5-79729
anti-Tuj1	Milliproe, mouse monoclonal antibody	1:200	MAB1637
anti-GFAP	Invitrogen, mouse monoclonal antibody	1:200	14-9892-82
anti-CREB5	Santa Cruz, mouse monoclonal antibody	IF: 1:50<br>WB: 1:200<br>ChIP: 1:300	sc-130435
anti-BrdU	Abcam, mouse monoclonal antibody	1:200	ab8152
Anti-Ki-67	Invitrogen, rat monoclonal antibody	1:1000	14-5698-80
anti-DCX	Abcam, rabbit polyclonal antibody	1:1000	ab18723
anti-NFIX	GeneTex, rabbit polyclonal antibody	1:500	GTX118630
anti-β-actin	Sigma-Aldrich, mouse monoclonal antibody	1:5000	A1978
anti-rabbit IgG (H+L) secondary antibody, Alexa Fluor 488	Invitrogen, goat polyclonal antibody	1:500	A-11008
anti-mouse IgG (H+L) secondary antibody, Alexa Fluor 594	Invitrogen, goat polyclonal antibody	1:500	A-11005
anti-mouse IgG (H+L) secondary antibody, Alexa Fluor 488	Invitrogen, goat polyclonal antibody	1:500	A-11001

anti-rat IgG (H+L) secondary antibody, Alexa Fluor 594	Invitrogen, goat polyclonal antibody	1:500	A-11007
HRP-conjugated anti-Mouse IgG	Sigma-Aldrich, goat polyclonal antibody	1:10000	AP130P
HRP-conjugated anti-rabbit IgG	Sigma-Aldrich, goat polyclonal antibody	1:10000	AP307P
